# Rethinking raptors: caracaras as a model for avian cognition in the wild

**DOI:** 10.1007/s10071-025-02007-3

**Published:** 2025-10-30

**Authors:** Katie J. Harrington, Laura M. Biondi, Megan L. Lambert

**Affiliations:** 1 Comparative Cognition Unit, Messerli Research Institute, University of Veterinary Medicine Vienna, Veterinärplatz 1, 1210 Vienna, Austria; 2 Instituto de Investigaciones Marinas y Costeras (IIMyC), UNMdP - CONICET, Juan B. Justo 2550, Mar del Plata, B7602GSD Argentina

**Keywords:** Behavioral flexibility, Cognitive ecology, Cognitive evolution, Comparative cognition, Innovation

## Abstract

Caracaras (subfamily Polyborinae) are a behaviorally distinctive and underexplored lineage of falcons that offer valuable opportunities to investigate the evolution of avian cognition in ecologically valid settings. Belonging to the sister clade to parrots and corvids—groups renowned for their cognitive abilities—caracaras express key traits linked to cognitive evolution, including large brains, high pallial neuron counts, complex social structures, ecological flexibility, and generalist foraging strategies. This review synthesizes existing behavioral, ecological, and neurological evidence suggesting that caracaras are well-suited for testing hypotheses in both social and physical cognition. Caracaras’ accessibility and behavioral responsiveness in the wild make them exceptionally well-suited for field-based studies, with the potential to address a critical gap in ecologically relevant comparative cognition. We highlight specific opportunities for research and emphasize that caracaras are a promising model for investigating how cognition evolves in response to varied socio-ecological pressures. We conclude that expanding cognitive research to include caracaras will broaden taxonomic representation and may offer broader insights into patterns of cognitive evolution.

**Supplementary Information:**

The online version contains supplementary material available at 10.1007/s10071-025-02007-3.

## Introduction

Cognitive abilities help animals to respond flexibly and adaptively to social and environmental challenges and can have both individual and population level consequences (Shettleworth 2009; Morand-Ferron and Quinn 2015; Huebner et al. 2018). Over the past several decades, comparative research with parrots and corvids has revealed cognitive parallels with primates, suggesting convergent evolution from shared socio-ecological pressures (Seed et al. 2009; Van Horik et al. 2012; Lambert et al. 2019). Despite growing incorporation of other species, our understanding of avian cognition still stems largely from these two avian groups, particularly in captivity (Emery 2006; Lambert et al. 2019). Expanding beyond cognitively well-studied parrots and corvids is essential for understanding how cognitive traits evolve and under what conditions (Van Horik et al. 2012; Lambert et al. 2022).

**Fig. 1:**
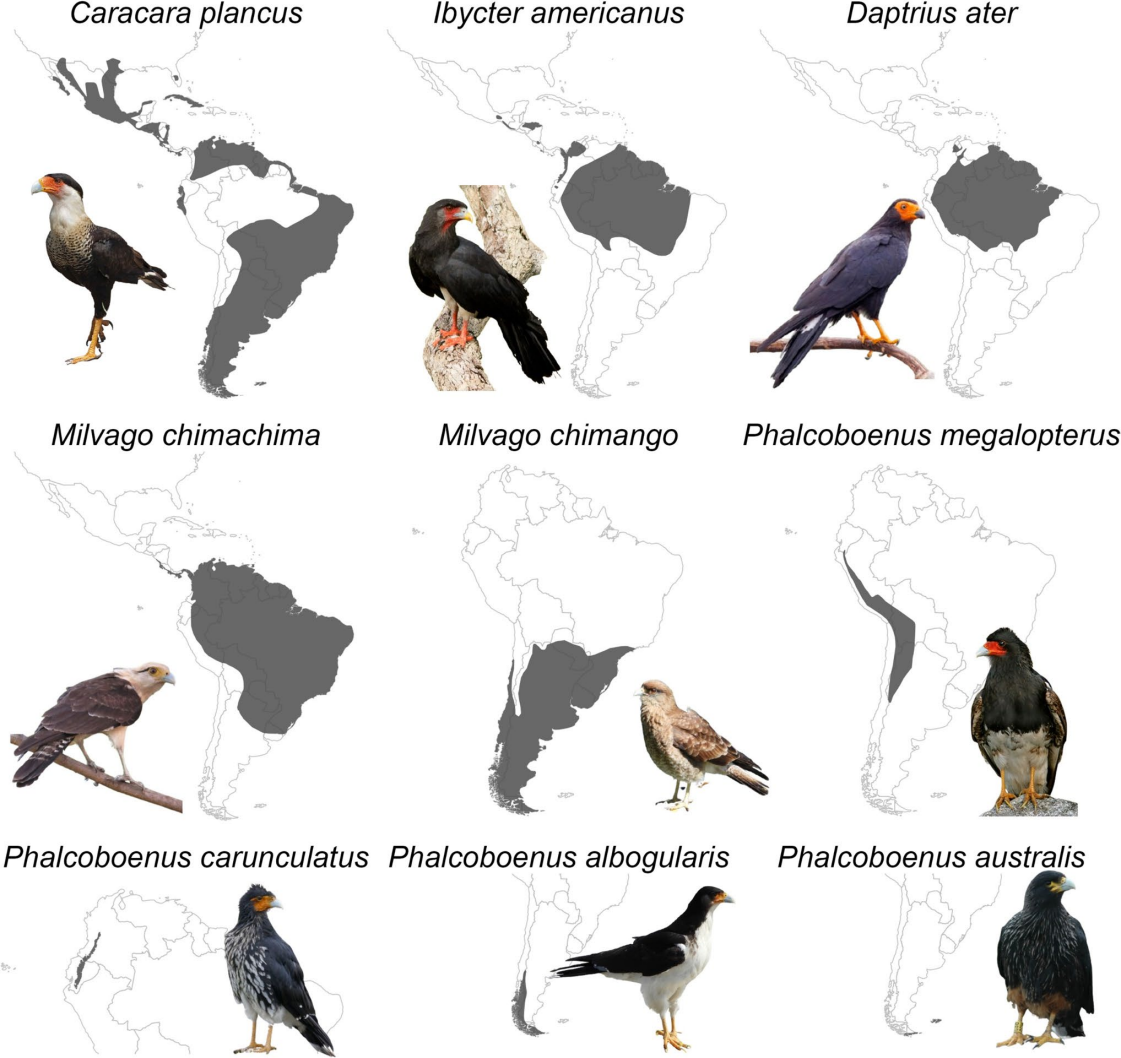
Extant caracara species and their distributions. Common names from left to right and top to bottom: crested caracaras (largest of the group), red-throated, and black caracaras; yellow-headed, chimango (smallest of the group), and mountain caracaras; and carunculated, white-throated, and striated caracaras. Photos are licensed under Creative Commons and credited from left to right and top to bottom: Joseph C Boone, Sean McCann, and Charles J. Sharp; felixú, Raf24 ~ commonswiki, and Elmarto; Rockel83, Raf24 ~ commonswiki, and Katie J. Harrington. Distribution maps from BirdLife International and Handbook of the Birds of the World (2024). See supplementary information for full photo credits

Falconiformes were recently established to be a sister clade to parrots and corvids, sharing a common ancestor roughly 66 MYA (Suh et al. 2011; Jarvis et al. 2014; Prum et al. 2015; Kumar et al. 2022). Despite this, Falconiformes have mostly been neglected in cognitive research, perhaps for their functional labeling as ‘birds of prey’: a group traditionally reputed as scarce, elusive, and difficult to study due to challenges with capture, handling, and intractability (Negro and Galván 2018; Biondi 2021). However, this overlooks a behaviorally distinct subfamily: the caracaras (Polyborinae), comprising nine extant species endemic to the Americas (Fig. 1, Table 1). Long viewed by falconers and naturalists as uniquely intelligent (Hudson 1920; Strange 1996), caracaras remain surprisingly understudied in cognitive research, with few exceptions (e.g., chimango caracaras, *Milvago chimango*: Biondi et al. 2010a, 2022; and striated caracaras, *Phalcoboenus australis*: Harrington et al. 2024a, b).

**Table 1:** Caracara species' cognitive ecology. Note: lack of information does not imply lack of trait.

Species	Range	Habitat	Foraging	Nonbreeding	Breeding	Cognitive studies
Crested caracara *Caracara plancus*	Central and South America, extending into North America	Grassland, savanna, agricultural, urban	Generalist, Predator, Scavenger, Kleptoparasitic, Fisher, Cacher, Extractive, Anthropogenic	Communal roosts, Scavenging aggregations	Monogamous	Associative learning (Potier et al. 2019)
Red-throated caracara *Ibycter americanus*	Southern Mexico, Central and South America	Tropical forest	Facultative specialist, Extractive, Cooperative	Group-living, Cooperative foraging	Cooperative	None known
Black caracara *Daptrius ater*	Amazon Basin	Lowland tropical forest, river edges	Generalist, Predator, Scavenger, Fisher, Cleaner	Small groups	Unknown	None known
Yellow-headed caracara *Milvago chimachima*	Central and northern South America	Savanna, lowlands, urban	Generalist, Predator, Scavenger, Fisher, Cleaner	Small groups	Unknown	None known
Chimango caracara *M. chimango*	Southern South America	Grassland, urban, agricultural	Generalist, Predator, Scavenger, Fisher, Kleptoparasitic, Cacher, Cleaner, Extractive, Anthropogenic	Communal roosts, Small to medium groups	Monogamous, Colonial and solitary nesting	Problem-solving, social learning, reversal learning, neophobia, object manipulation and exploration (Biondi et al. 2008, 2010a, b, 2013, 2015, 2020, 2022, 2024; Guido et al. 2017)
Mountain caracara *Phalcoboenus megalopterus*	Southern Ecuador, Chile, Argentina	Semi-desert puna plateau	Generalist, Scavenger, Cooperative	Unknown	Unknown	None known
Carunculated caracara *P. carunculatus*	Ecuador, Colombia	Páramo, alpine grasslands	Generalist	Small groups	Unknown	None known
White-throated caracara *P. albogularis*	Chile, Argentina	High mountain barren landscapes, herbaceous steppe	Generalist, Scavenger, Anthropogenic	Unknown	Unknown	None known
Striated caracara *P. australis*	Falkland Islands, southern South America	Subantarctic, coastal	Generalist, Predator, Scavenger, Kleptoparasitic, Extractive, Fisher, Cleaner, Cacher, Anthropogenic, Facultative nocturnal	Communal roosts, Scavenging aggregations, Fission–fusion	Monogamous, Cooperative care observed	Innovative problem solving, memory, object play (Harrington et al. 2024a, b; Harrington and Lambert 2024)

Caracaras exhibit several socioecological traits commonly linked to the evolution of cognitive abilities, including extended juvenile periods, complex social dynamics, and generalist foraging strategies (Strange 1996; Emery 2006; Sazima 2007; McCann et al. 2010). Unlike most Falconidae, they are predominantly non-migratory, nest-building, and long-legged terrestrial birds (Ferguson-Lees and Christie 2001; Mosto et al. 2016), with some species also displaying pronounced behavioral flexibility, gregariousness, and playfulness (Biondi et al. 2013, 2024; Harrington and Lambert 2024). Across the subfamily, caracara species have radiated into a wide range of environments, from southern subpolar islands and alpine puna to Amazon lowlands and anthropized grasslands (Fuchs et al. b^2012^; Morrison and Saggese 2024). Crested caracaras are a notable exception, spanning much of South and Central America, Cuba, Mexico, and the southern United States, and continuing to expand northward (Smith and Dwyer 2024), a geographic flexibility reminiscent of highly adaptable common ravens, noted for broad environmental tolerance and behavioral versatility (Garcia-Porta et al. 2022). Despite their ecological breadth, some species overlap locally at concentrated resources, frequently exploiting municipal dumps, farms, and fishing sites (Brown and Amadon 1968; Sazima 2007; Harrington et al. 2018; Bouker et al. 2021; Pantoja-Maggi et al. 2024), with some species even thriving in urban environments (Biondi et al. 2020; Lima et al. 2022).

In addition to socioecological traits, caracaras also exhibit neuroanatomical features associated with cognitive performance and technical innovation, including relatively large brains and high numbers of pallial neurons (Lefebvre et al. 1997; Nicolakakis and Lefebvre 2000; Sol et al. 2022). Compared to other Falconidae, and across birds more broadly, they rank among the highest in both absolute and relative brain size (Sol et al. 2022). Notably, striated caracaras possess approximately 663 million pallial neurons, placing them in the top 13.5% of 111 avian species surveyed by Sol et al. (2022), alongside cognitively renowned species such as common ravens (*Corvus corax*, 1.2 billion pallial neurons), African grey parrots (*Psittacus erithacu*s, 850 million), Goffin’s cockatoos (*Cacatua goffiniana*, 599 million), and western jackdaws (*Corvus monedula*, 491 million) (Sol et al. 2022). Interestingly, striated caracaras are part of a select group of birds including parrots, corvids, and owls, in which pallial neuron numbers outnumber those in the cerebellum (Gutiérrez-Ibáñez et al. 2025), a pattern that contrasts with most birds (including other Falconiformes), where cerebellar neurons typically dominate and are thought to play a key role in cognitive processing (Gutiérrez-Ibáñez et al. 2025). Further study is needed to determine whether this attribute is shared across other caracara species. These neuroanatomical traits suggest a valuable opportunity to explore how and why caracaras diverged from their falcon relatives and what such divergence may reveal about broader patterns of cognitive evolution in birds.

Given caracaras’ striking suite of socioecological and neuroanatomical traits, we propose a substantial opportunity to expand comparative cognitive research to include the caracara subfamily. Despite being understudied, many caracara species are behaviorally responsive, locally abundant, and relatively accessible in the wild—conditions that also offer rare opportunities to test cognitive hypotheses under natural settings. We organize our review around a framework spanning social and physical cognitive domains, by assessing current evidence for caracara cognition anchored in natural history, exploring relevant socioecological and evolutionary hypotheses, and identifying key avenues for future field-based research.

## Cognition across contexts

### Sociality

Prominent theories of cognitive evolution emphasize the challenges animals face in navigating social interactions (see Speechley et al. 2024 for review) and the mechanisms that allow individuals to succeed in competitive or cooperative interactions. These include abilities such as individual recognition, memory for past interactions and relationships, anticipation of future interactions, and social problem solving (Jolly 1966; Humphrey 1976; Seyfarth and Cheney 2015). Social complexity (see Kappeler 2019 for a review of how social complexity is defined) has been shown to be related to inhibitory control, where animals living in complex social environments need to inhibit their behaviors in the presence of more dominant individuals (Amici et al. 2008; Johnson-Ulrich and Holekamp 2020). As species’ social complexity increases, so might the selective pressure on individuals’ cognitive capabilities (i.e., social intelligence hypothesis, Jolly 1966; Humphrey 1976). Individuals of highly social species have been shown to be more proficient than more solitary species at discrimination, memory, and transitive inference (Bond et al. 2003; Hick et al. 2014), perhaps as these processes facilitate hierarchies, social bonds, cooperation, and competition. Group size has also been shown to positively correlate with the occurrence and spread of innovative behavior, possibly facilitated by increased opportunity for social information use (Ashton et al. 2018).

Caracaras exhibit a variety of social systems characterized by both traditional traits, such as fission–fusion dynamics and flexible cooperative breeding, and more recently emphasized quantitative metrics like the number and strength of differentiated relationships (Thiollay 1991; Strange 1996; Sazima 2007; Biondi et al. 2010b; Harrington and Meiburg 2021; for definitions, see Bergman and Beehner 2015; Boucherie et al. 2019), providing a valuable comparative framework for investigating the cognitive demands of social living. Some caracara species—such as the striated, crested (*Caracara plancus*), and chimango caracaras—form dynamic, temporary non-breeder groups prior to establishing monogamous adult pairs. These non-breeder groups roost communally (Johnson and Gilardi 1996; Josens et al. 2013; Harrington et al. 2018; Dwyer et al. 2018) and fluctuate in size, from few (e.g., milling and playing, Harrington and Lambert 2024) to thousands of individuals (e.g., aggregating at ephemeral resources or engaged in communal roosting, Josens et al. 2013; Tubelis 2019; Harrington et al. 2021). Carunculated caracaras have been observed foraging in groups of up to 40 individuals, usually near cattle or llamas; these may also represent non-breeder aggregations, although their age composition remains unknown (Ferguson-Lees and Christie 2001). This non-breeding period has been argued to be a critical aspect of avian social complexity driving the emergence of socio-cognitive skills, due to its importance for establishing and maintaining relationships and providing opportunities for social learning, competition, and cooperation (Boucherie et al. 2019). In support of this, there is evidence for social learning in chimango caracaras: wild-caught juveniles who observe trained demonstrators in a novel foraging task are significantly better than naïve individuals at the same task and furthermore outperform adult observers (Biondi et al. 2010b). Comparable evidence comes from yellow-headed caracaras (*Milvago chimachima*), where bathing behavior has been observed to spread among juveniles through horizontal social transmission (Vargas-Masís et al. 2014; De La Ossa V et al. 2018).

Caracaras present a compelling system for further investigating the characteristics of non-breeder social groups, such as persistence, social structure, and interactions (e.g., hierarchies, stable affiliations, and conflict support), as well as their role in socio-cognitive development (Boucherie et al. 2019). For example, as wild striated caracaras readily engage with experimental task apparatuses, one could study whether the number and strength of individuals’ differentiated relationships predicts performance in discrimination and memory tasks (Bergman and Beehner 2015). We can further ask whether communal roosting facilitates social information use as observed in common ravens (Wright et al. 2003); if individuals’ explorative nature may modulate reliance on social information as seen in kea parrots (*Nestor notabilis*, Huber et al. 2001; Suwandschieff et al. 2023); or how behavioral patterns or innovations may diffuse among fluctuating non-breeder groups (e.g., see Aplin 2016).

Beyond the nonbreeder stage, it has been proposed that mating systems may influence cognitive evolution. For example, the relationship intelligence hypothesis proposes that long-term pair bonds drive cognitive abilities, supported by findings that their prevalence correlates with larger brain size, presumably due to demands of synchrony, cooperation, and conflict resolution (Emery et al. 2007; though see Hooper 2021). Many caracara species appear to be monogamous and remain on their territories year-round (Morrison 1999; LMB personal observation; KJH unpublished data), with both sexes participating in nest-building, incubation, provisioning young, and nest defense (Morrison and Phillips 2000; Salvador 2016; Gallego-García et al. 2024; Ulises Balza unpublished data). Striated caracara pair members often remain in proximity (i.e., within 100 m), forage together, engage in vocal duets, and allopreen (Strange 1996; KJH unpublished data). However, in some cases, brood care extends beyond the pair, for example striated and chimango caracaras have been observed with three or four adults involved in parental care (Raimilla et al. 2014; LMB personal observation), a cooperative nesting strategy that, due to its reliance on social tolerance or proactive prosociality, may facilitate performance in socio-cognitive tasks (i.e., cooperative breeding hypothesis, see Burkart and van Schaik 2010, though also Burkart and van Schaik 2016 and Thornton and McAuliffe 2015). Interestingly, chimango caracaras furthermore nest both solitarily and colonially, sometimes within the same population (Fraga and Salvador 1986), offering a natural context to examine how breeding structure shapes the use of social information in decision-making (Evans et al. 2016) and the emergence of prosociality (Horn et al. 2020). Red-throated caracaras (*Ibycter americanus*), on the other hand, are obligate cooperative brooders (e.g., 5–7 individuals per brood), and display a range of task sharing, including nest guarding, brood care, allofeeding, and coordinated foraging with vocally maintained cohesion (Thiollay 1991). The diversity of mating systems and social breeding structures across caracaras provides a valuable framework to test how different reproductive strategies shape the development and expression of socio-cognitive traits, such as coordination, role differentiation, and social learning, in naturalistic settings.

More broadly, sociality enables cooperative behaviors such as task sharing, cooperation, and division of labor, which may be cognitively demanding by requiring individual recognition, coordination, and the ability to manage delayed rewards (Brosnan et al. 2010). Across caracara species, cooperative behavior occurs in both pair and group contexts, offering varied opportunities to investigate the cognitive mechanisms that may underlie joint action. Mountain caracaras (*Phalcoboenus megalopterus*), for example, have been observed cooperatively overturning rocks while foraging for invertebrates (Jones 1999). Striated and chimango caracaras form dynamic alliances to procure and defend food resources and may use distraction tactics to allow others to access unattended prey (e.g., striated caracaras obtaining seabird eggs or vying for carrion that has washed ashore; chimango caracaras capturing pigeons; Strange 1996; KJH unpublished data; LMB personal observation). Crested caracara adult pairs appear to coordinate hunting (Whitacre et al. 1982), and aside from foraging, have been observed engaging in interspecific allopreening (a form of cooperative behavior) with black vultures (*Coragyps atratus*) in the Brazilian Pantanal (Palmeira (Lopes Palmeira, 2008)); chimango caracaras form mobbing alliances for collective defense, which in turn result in food facilitation events (Baladrón et al. 2009); and red-throated caracaras engage in food sharing among adult group members, delivered over distance (e.g., a piece of wasp nest; Thiollay 1991). Red-throated caracaras also forage cooperatively in groups of five to seven, comprised of feeding birds, attendants, and apparent sentinels (Thiollay 1991; McCann et al. 2013), which may require coordination and partner monitoring, skills potentially facilitated by memory, learning, and decision-making (Brosnan et al. 2010). Although empirical data are limited, their cooperative foraging patterns (in addition to their cooperative nest defense) suggest potential for social learning and role specialization. Studying such group dynamics may reveal how cognitive demands differ between solitary and group-foraging species. These varied examples also offer a comparative framework for exploring whether similar cognitive processes underlie cooperation across domains. Furthermore, understanding these social capabilities and their potential benefits is essential for predicting how environmental or ecological constraints might limit group formation or the expression of social behaviors.

Beyond cooperation, social species must also manage conflict, often through dominance hierarchies that regulate access to resources and shape social interactions (Drews 1993; de Waal 2000). Maintaining such hierarchies may depend on cognitive skills like self or mutual assessment, transitive inference, and tracking a broad network of individuals and interactions (Tibbetts et al. 2022). Striated and crested caracaras appear to follow dominance hierarchies with adults controlling access to resources (Dwyer and Cockwell 2011; Autilio et al. 2019); in striated caracaras, subordinates have been observed using appeasement signals (e.g., hunched display and begging calls) or conspecific recruitment (e.g., repeated vocalization) to navigate these interactions (Harrington and Meiburg 2021)—a pattern reminiscent of hierarchical corvids and parrots like common ravens and kea, where subordinates employ mixed appeasement and aggression signals to increase tolerance from dominants (Heinrich 1988; Diamond and Bond 1991). In contrast, dominant adult striated caracaras tolerate younger individuals when resources are abundant (e.g., common terrestrial invertebrates), foraging alongside and allowing displacement, which provides an opportunity to investigate the role of tolerance in social learning, as it has recently been shown to underlie cultural transmission of novel foraging behaviors (Coelho et al. 2024). These behaviors raise questions about the form (e.g., linear vs. non-linear) and stability of caracara hierarchies; if individuals can monitor rank relationships, as observed in common ravens (Massen et al. 2014); and if rank predicts performance on tasks related to social monitoring, memory, or decision-making (Hobson and DeDeo 2015; Snijders et al. 2017; Prasher et al. 2019; Milewski et al. 2022).

### Foraging

In addition to social demands, the universal challenge of acquiring food has been proposed as a key driver of cognitive evolution, shaped by factors such as dietary generalism, resource variability, and foraging strategy (Parker and Gibson 1977; Overington et al. 2008; Rosati 2017). Across taxa, dietary breadth is associated with larger brains and higher technical innovation rates, likely because generalists must flexibly track changing resources (Ducatez et al. 2015).

Caracaras are dietary generalists, opportunistically consuming a broad range of foods, including carrion, small birds and mammals, reptiles, eggs, marine and terrestrial invertebrates, palm fruits, and anthropogenic food types (Haverschmidt 1962; Biondi et al. 2005; Sazima 2007; Rexer-Huber and Bildstein 2013; De La Ossa-V et al. 2018; Carranza and Anderson 2019; Harrington and Bildstein 2019; de Godoy et al. 2020; Bouker et al. 2021; Pantoja-Maggi et al. 2024; Balza et al. 2020). Yellow-headed caracaras further exploit ephemeral opportunities such as insect emergences (e.g., reproductive alates of Atta sp., Camacho et al. 2012). Several species, including black (*Daptrius ater*), yellow-headed, crested, chimango, and striated caracaras, also facultatively engage in cleaning mutualism, consuming ectoparasites from mammalian hosts such as tapirs (Tapirus spp.), capybaras (*Hydrochoerus hydrochaeris*), sloths (*Bradypus variegatus*), and livestock (Peres 1996; Krakauer 1999; Ferguson-Lees and Christie 2001; Sazima and Sazima 2010a, b; Gijsman and Guevara 2020; KJH personal observation), a strategy that may rely on cognitive mechanisms such as individual recognition, social memory, and decision-making, as suggested in other taxa (Soares 2017).

The predominance of dietary generalism among caracaras offers a useful baseline for assessing how other ecological or social factors—such as habitat breadth, resource fluctuations, or group structure—may shape cognitive variation. Additionally, examining finer-scale variation in dietary breadth across species or seasons, including niche shifts and nutritional composition, may clarify how nutritional ecology influences cognitive performance (Machovsky-Capuska et al. 2016).

One notable exception to caracaras’ dietary generalism is red-throated caracaras, which facultatively specialize in wasp and bee broods (Thiollay 1991; McCann et al. 2013). This species may therefore serve as a case study on the correlation between dietary specialism and morphological, behavioral, or cognitive traits, in contrast to the more dietary generalist caracaras. One could furthermore contrast the cognitive demands of predatory specialism versus predatory generalism, the latter of which may require greater cognitive flexibility in tracking, capturing, and handling a variety of live prey (e.g., lizards, birds, fish) than scavenging alone (Swanson et al. 2012; Bailey et al. 2013).

Fluctuating resource availability, for example due to seasonality and human behavior, may also select for cognitive flexibility by favoring strategies like caching, resource-switching, or innovation. Among caracaras, exposure to resource fluctuation varies by geography and proximity to human activity. For example, mountain and striated caracaras face seasonally mediated foraging limitations such as snow cover and pulsed resources associated with migratory prey (Strange 1996; Donadio et al. 2007; Rexer-Huber and Bildstein 2013; Balza et al. 2020). Crested caracaras offer a unique opportunity to investigate the effect of fluctuating resources within species, as the Florida (USA) population has access to continuously available year-round resources (e.g., those associated with agricultural practices), while the Falkland Islands (Islas Malvinas) population—similar to striated caracaras—faces the limitations of migratory prey (Catry et al. 2008).

Caching, as a strategy for coping with resource fluctuations, may rely on cognitive processes such as spatial memory, content tracking, temporal discrimination, and possibly cache monitoring, skills that have made caching species valuable models for studying memory and decision-making (Grodzinski and Clayton 2010; Sonnenberg et al. 2019). Similar to other falcons and birds of prey (Holthuijzen 1990; Cameron and Olsen 1993; Kerr 1999; Charley et al. 2014; Fitzsimons et al. 2019), many caracaras facultatively cache, including crested, chimango, striated and red-throated caracaras, although the frequency, context, and mechanisms of this behavior remain poorly understood (Strange 1996; Bennett et al. 2014; Woods et al. 2017; Morrison and Dwyer 2023; LMB personal observation). In chimango caracaras, caching observations occur mostly during the breeding period, when food demand and intraspecific kleptoparasitism are highest (LMB personal observation). Given findings in corvids that pilfering risk drives re-caching and cache protection strategies (Grodzinski and Clayton 2010), caracaras provide a promising system for exploring whether similar pressures shape caching cognition in facultative cachers.

Movement data may further illuminate cognitive responses to resource variability. Juvenile striated caracaras in the Falkland Islands (Islas Malvinas) travel repeatedly (i.e., on successive days) to ephemeral food sources, suggesting memory-based spatial use patterns (Gautestad et al. 2013; Harrington et al. 2018, 2020), consistent with growing evidence across birds and mammals that animal movement can be shaped by cognitive processes such as memory and decision-making (see Kashetsky et al. 2021 for review). Furthermore, nonbreeding striated caracaras in the Falklands (Malvinas) adjust their range seasonally, moving between seabird colonies in summer and human settlements in winter (Harrington et al. 2018). These shifts can alter social density, competition, and energetic tradeoffs, offering a natural framework to test whether cognitive performance varies across seasons in tasks related to resource acquisition (e.g., motor self-regulation, memory, or learning tasks) or social cognition (e.g., social tolerance, cooperation, social learning) (see Rochais et al. 2021 for an example in mammals).

Relatedly, several caracara species appear to anticipate the timing of food availability, positioning themselves at predictable resource sites, such as birthing marine mammals and turtles or human activity zones like trash collection points (Strange 1996; Sazima 2007; Harrington et al. 2018). In urban and rural contexts, this includes the possibility of learning temporal human activity patterns, such as associating specific times of day or regular schedules of waste disposal with reliable feeding opportunities, and potentially recognizing individuals involved in these activities. These behaviors may reflect simple associative learning based on time-place regularities—a form of learning fundamental to decision making—or more complex spatiotemporal memory akin to episodic-like memory reported in other birds (Shettleworth 2009).

Extractive foraging for food that is not directly perceptible has also been posited as a potential avenue for the convergent evolution of cognition in corvids and apes, as it requires sensorimotor control, object manipulation, and mental representation (i.e., sensorimotor intelligence hypothesis; Parker and Gibson 1977, 1979; Seed et al. 2009; O’Hara and Auersperg 2017). Comparative analyses in woodpeckers provide additional support for this view beyond these two taxa, showing that species specializing on wood-boring larvae tend to have relatively larger brains, with such behaviors likely arising in lineages that retained large ancestral brains (Cárdenas-Posada et al. 2023). Several parrot and corvid species extract food, and for some, this constitutes a major part of their diet (Lockie 1955; Diamond and Bond 1999; Rutz et al. 2010; Mioduszewska et al. 2022). Caracaras use a range of extractive foraging techniques (e.g., excavating invertebrates, dismantling desiccated root systems, extracting larvae and eggs from nests, and opening garbage bags; (Thiollay 1991; Sazima 2007; Harrington et al. 2021; LMB personal observation). Similar to little ravens (*Corvus mellori*), striated caracaras also enter Magellanic penguin (*Spheniscus magellanicus*) burrows to retrieve concealed prey and carrion (Ekanayake et al. 2015, KJH personal observation), which may qualify as extractive foraging, as it requires locating and accessing food that is not directly visible. Red-throated caracaras, on the other hand, extract larvae and eggs from wasp and bee nests and furthermore peel fruits such as umari sauvage (*Poraqueiba guianensis*, Thiollay 1991; McCann et al. 2013). The various extractive foraging techniques allow for wider comparisons about how motor dexterity (e.g., hindlimb use in grasping and bringing objects to the beak) and laterality may influence both problem-solving capacity and physical cognition across species (Magat and Brown 2009; Biondi et al. 2022; Gutiérrez-Ibáñez et al. 2023; Colbourne et al. 2024). The behavior of entering burrows suggests an ecologically grounded system for testing object permanence in wild falcons. Given that stage 4 object permanence (visible displacement) is widespread across taxa (Cacchione and Rakoczy 2017; Schaffer et al. 2024), it is reasonable to assume that caracaras possess this basic capacity. The more open question is whether they, like some corvids and parrots, also succeed at higher stages such as invisible displacement (Cornero and Clayton 2025).

Kleptoparasitism (taking food procured by another individual) may rely more on cognitive skills than physical strength, such as identifying social cues, decision time, and possible planning and avoidance (Morand-Ferron et al. 2007; Gallego-Abenza et al. 2020). In birds, kleptoparasitic species tend to have larger residual brain size compared to their respective hosts (Morand-Ferron et al. 2007) and increased fitness (Shealer et al. 2005). While the cognitive skills facilitating the behavior have been little studied, in common ravens, decision time (i.e., to fly off with food or consume in situ) inversely relates to avoidance of kleptoparasitism (Gallego-Abenza et al. 2020). Crested, chimango, and striated caracaras kleptoparasitize both conspecifics and heterospecifics (García and Biondi 2011; Partida and Rodríguez-Estrella 2015; KJH personal observation). Investigating this behavior provides an opportunity to identify tactical strategies, avoidance behaviors, and the cognitive mechanisms that may support them.

While many of the foraging strategies discussed above likely reflect species-typical behaviors adapted to particular ecological contexts, animals may also face situations where established tactics are insufficient. In these cases, innovation—generating novel solutions to novel or existing problems—may become a critical means of coping with change (Kummer and Goodall 1985; Reader and Laland 2003). Behavioral innovations can enable animals to adaptively respond to changing conditions by sampling novel foods or developing new search and handling techniques in unfamiliar contexts (Overington et al. 2009; Ducatez et al. 2015). In parrots and corvids, innovation has been linked to larger relative brain size and greater ecological flexibility (Lefebvre et al. 2004; Overington et al. 2009). While anecdotal reports are common, only more recently have researchers begun empirically testing innovative behavior in birds under natural conditions, which is critical for understanding how cognition contributes to fitness in ecologically valid settings (Lee and Thornton 2021; Szabo et al. 2022). Recent studies highlight the potential of caracaras for this purpose. Wild striated caracaras voluntarily and rapidly solve novel extractive foraging tasks and retain solution techniques across years without reinforcement (Harrington et al. 2024a, b). As striated caracaras experience seasonally pulsed resources, they offer further opportunities to test hypotheses for the emergence of innovation in the wild (e.g., the bad competitor hypothesis or the excess of energy hypothesis, see Amici et al. 2019 for a review) according to seasonal resource abundance and individual foraging success and body condition. Captive-tested wild chimango caracaras also show behavioral flexibility and rapid learning in puzzle-box and detour tasks (Biondi et al. 2010a, 2024), capacities which were more pronounced in individuals that exploit urban environments. Together, these findings suggest that caracaras, particularly striated and chimango species, offer a rare opportunity to investigate cognitive innovation in free-ranging birds using ecologically relevant paradigms.

### Beyond foraging

The long-standing view that nest building is a rigid, instinctive behavior has been contradicted by recent comparative research demonstrating intraspecific variation and behavioral flexibility in how birds construct their nests (Perez et al. 2023; Lehtonen et al. 2023). This flexibility underscores the argument that nest building may play a significant role in the emergence of physical cognition, as it requires individuals to be selective in when, where, and how to choose sites and materials (Guillette and Healy 2015). Unlike most falcons (e.g., genus *Falco*) which do not typically build nests, instead for example, using other species’ nests or laying eggs in shallow crevices (Del Hoyo et al. 1992), some caracara species build, maintain and defend nests. Crested caracaras select materials such as twigs, livestock hairs, bones, and grass to construct large and highly visible nests (Liébana et al. 2024a). They have also begun nesting on man-made structures and incorporating man-made materials, such as fabrics, plastics, wire, and twine (De Lucca and De Lucca 2017; Saggese et al. 2022; Lima et al. 2022; Liébana et al. 2024a). White-throated caracaras build nests using similar materials and construction, though located on ledges or in crevices or caves (Grande et al. 2024). On the other hand, red-throated caracaras do not seem to build nests but rather use natural platforms like bromeliads (McCann et al. 2010, though see exception Bennett et al. 2014). Yellow-headed caracaras have been documented adopting existing, man-made structures such as oil cans, buckets of corn or wooden boxes for their nesting, though will also build stick nests in trees (Johansson et al. 1999; Ferguson-Lees and Christie 2001). Similarly, chimango caracaras build nests in trees, on the ground over low vegetation and on anthropogenic structures (Ferguson-Lees and Christie 2001; Biondi et al. 2005; Liébana et al. 2024b). They also use natural and anthropogenic cavities for nesting (De Lucca et al. 2022; Galmes et al. 2024). Caracaras’ interspecies variation in site selection, construction, and maintenance provides an exciting opportunity to test hypotheses regarding the role of nest building—particularly of increased object manipulation and establishment of relationships between objects—in physical cognition. Furthermore, these species-specific nesting behaviors, especially material choice and object manipulation, may serve as naturalistic expressions of physical cognition and motor planning—parallel to tool selection in other taxa (Hansell and Ruxton 2008).

Exploratory and playful tendencies may also translate to enhanced physical cognition through increased learning opportunities (Call 2013; Auersperg 2015). Among caracaras, chimango and striated of all ages (though most pronounced in juveniles) stand out for their exploratory tendencies, showing an interest in novelty that rivals highly explorative kea parrots (Biondi et al. 2010a, 2013; Harrington and Lambert 2024). Both chimango and striated caracaras repeatedly and voluntarily interact with natural and man-made novel objects spontaneously and in experimental designs. Chimango caracaras engage for longer with objects of greater geometric complexity, possibly due to the potential for information gain (Biondi et al. 2013, 2015). Similarly, striated caracaras show a strong preference for objects with more pronounced movement feedback and tend to exhibit clear markers of object play, including rolling while grasping items with their feet (Harrington and Lambert 2024), as is often observed in corvids and parrots (Diamond and Bond 2004). Interestingly, kea parrots show increased exploration toward specific objects or features of an apparatus after watching a conspecific solve a task but quickly abandon social information in favor of exploration (Suwandschieff et al. 2023). Examining whether chimango and striated caracaras demonstrate a similar reliance on haptic exploration rather than visual information would provide insight into how these birds gather information and approach new challenges in the wild. Notably, as exploratory tendencies, perhaps due to differing levels of neophobia, vary between the Falklands (Malvinas) and mainland South American populations of striated caracaras (Ulises Balza personal communication), this also presents an opportunity to investigate the effects of insularity (i.e., island syndrome) on how individuals gather and process information (Gavriilidi et al. 2022).

### Domain general

Some cognitive abilities are thought to span socioecological contexts. These domain-general mechanisms, for example executive functions such as inhibitory control, flexible learning, and working memory, support behavioral adaptation across diverse challenges and are often assessed using tasks like reversal learning and detour-reaching, which offer standardized tools for comparing cognitive flexibility across species (Bond et al. 2007). In reversal learning tasks, captive-tested wild chimango caracaras perform comparably to parrots and corvids, which may facilitate their success in urbanized and rapidly changing environments (Guido et al. 2017; Biondi et al. 2024). In detour-reaching tasks, captive-tested adult chimangos show higher success than macaws tested under similar conditions, indicating more efficient learning in certain contexts (LMB unpublished data; Kabadayi et al. 2017). Chimango caracaras’ high exploratory tendencies (Biondi et al. 2010a, 2015) further support their suitability for testing hypotheses about the relationship between exploration and domain-general cognitive abilities, traits that may co-vary positively, or trade off if increased exploration reflects impulsivity and reduced behavioral inhibition (Griffin et al. 2013). Furthermore, recent findings suggest that domain-general performance (e.g., in reversal learning) can be modulated by behavioral traits such as neophobia: in chimangos, higher neophobia was linked to poorer flexibility, particularly in rural and suburban individuals, highlighting how individual and environmental variation may shape the expression of domain-general cognition (Biondi et al. 2024).

### Comparative perspectives within Falconidae

While comparisons among caracara species are especially informative for linking ecology and cognition, extending tests to other falcons would provide a valuable phylogenetically proximate outgroup. The most tractable starting points may be species of the genus *Falco*, which—owing to their long history in falconry and extensive captive or conservation programs—are particularly accessible for standardized testing. In wild populations, some generalist kestrels (American *F. sparverius,* Eurasian *F. tinnunculus*, and Lesser *F. naumanni*) are widely monitored through nestbox schemes in North America and Europe, providing existing infrastructure for repeated access to individuals. In captive settings, generalist peregrine falcons (*F. peregrinus*), long central to falconry traditions, could offer another feasible model for cognitive testing. Fargallo et al. (2022) provide a comprehensive dataset on trophic niche, geographic range size, and biomic specialization across Falconidae, which offers a valuable foundation for selecting species and framing comparative predictions. Together, such comparisons could provide ecologically and socially contrasting outgroups to the opportunistic and gregarious caracaras while minimizing phylogenetic distance, helping to disentangle the relative contributions of foraging strategy, sociality, and habitat use to cognitive evolution across Falconidae.

## Caracaras in the Anthropocene

As human activity continues to reshape ecosystems globally, caracaras, like many other species, face novel challenges beyond those posed by their evolutionary history. Crested and chimango caracaras have become increasingly common in urban areas (Leveau et al. 2022; Smith and Dwyer 2024), as well as white-throated and yellow-headed caracaras to a lesser extent (De La Ossa V et al. 2018; Grande et al. 2024). Recent evidence from citizen science illustrates how human activity influences caracara ecology even in remote or mountainous regions. Pantoja-Maggi et al. (2024) found that mountain and white-throated caracaras now frequently feed on garbage, carrion from domestic animals, and anthropogenic food, and in La Paz, Bolivia, mountain caracaras are increasingly present in urban environments where they exploit refuse, markets, and even prey on domestic pigeons (Richard and Contreras Zapata 2015).

Urban environments are cognitively demanding in ways that differ from natural habitats: animals must process a high volume of novel stimuli, discern patterns in resource distribution, and update behavioral strategies in response to fast-paced environmental changes. Lee and Thornton (2021) emphasize that such environments impose unique informational challenges, favoring individuals capable of flexible learning, categorization of novel cues, and effective decision-making. This is particularly relevant to generalist species like caracaras, who already exhibit opportunism and exploratory foraging. Notably, urban populations of chimango caracaras exhibit reduced neophobia, increased exploration and boldness, faster learning rates, and enhanced problem-solving abilities relative to rural conspecifics (Solaro and Sarasola 2019; Biondi et al. 2020, 2022, 2024). These findings echo a growing body of work suggesting that successful urban adaptation may hinge on cognitive and behavioral flexibility—a view consistent with the cognitive buffer hypothesis, which posits that cognitive abilities facilitate behaviorally adaptive responses to rapidly changing conditions (Allman et al. 1993; Allman 2000; Sol 2009; Lee and Thornton 2021).

Understanding how caracaras navigate the Anthropocene is critical for both conservation and cognition research. Studying how these birds respond to ecological novelty can inform the design of interventions aimed at mitigating human-wildlife conflict and promoting coexistence (Marzluff and Swift 2017; Goumas et al. 2020; Plotnik and Jacobson 2022). Moreover, urban and human-modified environments offer a natural experiment to investigate how cognitive traits, such as innovation, memory, and social learning, may allow species to adapt to rapid environmental changes.

## Conclusion

This review highlights caracaras as a compelling but underutilized model for advancing comparative cognition. Their behavioral diversity, ecological plasticity, and evolutionary ties to cognitively well-studied avian taxa offer rare opportunities to test longstanding hypotheses across both social and physical cognitive domains. Crucially, their accessibility in wild settings enables ecologically valid research designs that can address how cognition evolves and operates under natural conditions.

A strong grounding in species-specific natural history is essential for designing rigorous cognitive experiments (Thornton and Truskanov 2022). Morrison and Saggese (2024) provide a valuable synthesis of caracara research to date, helping identify underexplored species and guiding future research priorities. Nonetheless, much of the available literature remains concentrated on only a few of the nine extant species, reflecting both uneven research effort and possibly limited accessibility of studies across languages. While we surveyed the literature broadly, valuable information published in regional or local outlets can be difficult to identify and access, since these sources often lack visibility in international databases (Stergiou and Tsikliras 2006; Devenish-Nelson et al. 2017). This is exacerbated by the strong dominance of English in global scientific publishing, which can marginalize non-English contributions (Tijssen et al. 2006). Addressing these gaps through targeted field studies and improving the discoverability and integration of regional-language literature will be essential for providing the baseline knowledge needed for robust comparative cognitive research in caracaras. In combination with predictive modeling, researchers can generate targeted, ecologically grounded hypotheses about how cognition evolves in response to social and environmental pressures.

Moreover, evidence indicates that species typically described as having advanced cognitive abilities, such as parrots and corvids, often face greater welfare challenges in captivity (Mellor et al. 2021). Studying cognition in caracaras under natural conditions can inform rehabilitation, conservation and education programs, particularly for individuals that must remain in captivity (Mellor et al. 2021; Clark 2022). Insights from field-based research can also guide the development of cognitive enrichment to enhance welfare in these settings. By prioritizing studies of cognition in the wild, researchers can both deepen our understanding of caracara cognition and create applied tools that benefit conservation and animal welfare, ensuring that cognitive research serves both scientific and practical ends.

## Supplementary Information

Below is the link to the electronic supplementary material.

Supplementary Material 1

## Data Availability

No datasets were generated or analysed during the current study.
