# Supplementary material for: Rethinking raptors: caracaras as a model for avian cognition in the wild
Source: Anim Cogn. 2025 Oct 30;28(1):89. doi: 10.1007/s10071-025-02007-3 (PMC12575590; doi:10.1007/s10071-025-02007-3)
Supplement: Supplementary file 1 — Supplementary Material 1 [file 10071_2025_2007_MOESM1_ESM.docx]

Supplementary Material:

-Sample of online videos illustrating relevant observations

-Complete photo credits for Figure 1

**Sample of online videos illustrating relevant observations**

**Striated caracaras**

**Puzzle box**, wild, Falkland Islands

[@Cell Press]. (2024, Jan 8). Curious caracaras: The puzzle-solving wild falcons / Curr. Biol., Nov. 20, 2023. [Video]. YouTube.

**Voice commands**, captive, Woodlands Family Theme Park

[@Woodlands Family Theme Park]. (2008, Aug 26). Clever Bird. [Video]. YouTube. https://www.youtube.com/watch?v=K_QUlpdoFu0

**Puzzle feeder**, captive, *Woburn Safari Park, England*

[@TeamBuildingwithBITE]. (2023, July 7). Caracara puzzle feeder – complex enrichment for intelligent species. [Video]. YouTube. <https://youtu.be/StibQFr0tYM?si=suDVhvoutldi1I8p>

**Agility course**, captive, *Astur Falconry, Slovakia*

[@asturfalconry]. (2024, Dec 26). Our Benji starred again… [Video]. Instagram. https://www.instagram.com/reel/DECFKO5Ni96

**Voice commands**, captive, *Elite Falconry, Scotland*

[@elitefalconry]. (2023, Dec 3). Chaos the Caracara. [Video]. Facebook. <https://www.facebook.com/share/v/1Bga5nf3AS/>

**Crested caracaras**

**Puzzle feeder**, Suffolk Owl Sanctuary

[@Suffolk Owl Sanctuary]. (2024, Nov 3). Fred our Crested Caracara is very intelligent… [Video]. Facebook. <https://www.facebook.com/share/v/1BUPQTEqJM/>

**Habituation, feeding, low neophobia**, wild, Brazil

[@clovis.sm]. (2023, July 5). Unnamed. TikTok. <https://www.tiktok.com/@clovis.sm/video/7252437439346806021>

**Curiosity, low neophobia**, wild, Argentina

[@tripinargentina]. (2023, Dec 11). Increíble o que… [Video]. TikTok. https://www.tiktok.com/@tripinargentina/video/7311422247615417605

**Yellow-headed caracaras**

**Vagrant, foraging on anthropogenic resources**, Florida, United States

Manfredi, Larry. (2024, Jan 6). Yellow-headed caracara. Video. McCaulay Library ML613194010. <https://search.macaulaylibrary.org/catalog?taxonCode=yehcar1&mediaType=video>

**Feeding on palm nut**, Columbia.

Jimenez, Daniel. (2014, Nov 23). Yellow-headed caracara. [Video]. McCaulay Library ML201949131. <https://search.macaulaylibrary.org/catalog?taxonCode=yehcar1&mediaType=video>

**Crested, chimango, and white-throated caracaras**

**Foraging**, Ushuaia, Argentina

Iron, Jean. (2025, Jan 28). White-throated Caracara. [Video]. McCaulay Library ML633578302. <https://search.macaulaylibrary.org/catalog?taxonCode=whtcar1&mediaType=video>

**Complete photo credits for Figure 1**

*Authors removed background from all photos for use in Figure 1.*

"Crested Caracara JCB" by Joseph C Boone is licensed under CC BY-SA 3.0. https://openverse.org/image/7a466b97-8804-4e20-9db9-549846039a5e?q=crested+caracara&p=2

"Red-throated Caracara" by Sean McCann is licensed under CC BY-SA 3.0. https://openverse.org/image/d0c26b3f-ad28-4eda-af11-191552b77794?q=red-throated+caracara&p=1

"Black caracara (Daptrius ater) Rio Napo" by Charles J. Sharp is licensed under CC BY-SA 4.0. https://openverse.org/image/183427be-f95e-4c9c-9099-5cf6a4ab15a3?q=black+caracara&p=4

"Milvago chimachima / Pigua / Yellow-headed Caracara" by felixú is licensed under CC BY-SA 2.0. https://openverse.org/image/b377d2e8-e245-4323-b888-64a206675fb3?q=yellow-headed+caracara&p=27

"Milvago chimango, Nahuel Huapi" by Raf24~commonswiki is licensed under CC BY 4.0. https://openverse.org/image/5b9698b5-127a-4224-8f1f-fe93732c6fff?q=milvago+chimango&p=39

"Mountain caracara" by User:Elmarto is licensed under CC BY 3.0. https://openverse.org/image/945f3842-39d8-489d-97c3-374659efebd2?q=mountain+caracara&p=1

"Carunculated caracara 2" by Rockel83 is licensed under CC BY-SA 4.0. https://openverse.org/image/d3887f11-8c2a-4013-b0d0-5eeec807f8de?q=carunculated+caracara&p=4

"Phalcoboenus albogularis, Los Glaciares (cropped)" by Raf24~commonswiki is licensed under CC BY 4.0. https://openverse.org/image/5644eba5-75b4-41f8-a2e4-dccfd719b46f?q=white-throated+caracara&p=17

“Striated caracara on Saunders Island” by Katie J. Harrington.
